# The *Meloidogyne incognita* Nuclear Effector MiEFF1 Interacts With *Arabidopsis* Cytosolic Glyceraldehyde-3-Phosphate Dehydrogenases to Promote Parasitism

**DOI:** 10.3389/fpls.2021.641480

**Published:** 2021-04-09

**Authors:** Nhat My Truong, Yongpan Chen, Joffrey Mejias, Salomé Soulé, Karine Mulet, Maëlle Jaouannet, Stéphanie Jaubert-Possamai, Shinichiro Sawa, Pierre Abad, Bruno Favery, Michaël Quentin

**Affiliations:** ^1^Institut Sophia Agrobiotech, INRAE, CNRS, Université Côte d’Azur, Sophia Antipolis, France; ^2^Graduate School of Science and Technology, Kumamoto University, Kumamoto, Japan; ^3^Department of Plant Pathology and Key Laboratory of Pest Monitoring and Green Management of the Ministry of Agriculture, China Agricultural University, Beijing, China

**Keywords:** root-knot nematode (*Meloidogyne incognita*), effector, giant cell, cytosolic glyceraldehyde-3-phosphate dehydrogenases, susceptibility gene

## Abstract

Root-knot nematodes are obligate endoparasites that maintain a biotrophic relationship with their hosts over a period of several weeks. They induce the differentiation of root cells into specialized multinucleate hypertrophied feeding cells known as giant cells. Nematode effectors synthesized in the esophageal glands and injected into the plant tissue through the syringe-like stylet play a key role in giant cell ontogenesis. The *Meloidogyne incognita* MiEFF1 is one of the rare effectors of phytopathogenic nematodes to have been located *in vivo* in feeding cells. This effector specifically targets the giant cell nuclei. We investigated the *Arabidopsis* functions modulated by this effector, by using a yeast two-hybrid approach to identify its host targets. We characterized a universal stress protein (USP) and cytosolic glyceraldehyde-3-phosphate dehydrogenases (GAPCs) as the targets of MiEFF1. We validated the interaction of MiEFF1 with these host targets in the plant cell nucleus, by bimolecular fluorescence complementation (BiFC). A functional analysis with *Arabidopsis* GUS reporter lines and knockout mutant lines showed that GAPCs were induced in giant cells and that their non-metabolic functions were required for root-knot nematode infection. These susceptibility factors are potentially interesting targets for the development of new root-knot nematode control strategies.

## Introduction

Root-knot nematodes (RKNs), *Meloidogyne* spp., are extremely polyphagous biotrophic plant parasites with a worldwide distribution. These microscopic worms cause dramatic root deformations, known as galls or root knots, which decrease crop yields, resulting in considerable economic losses (Abad et al., 2008; Jones et al., 2013). RKN are obligate parasites with a life cycle of 3–8 weeks, depending on the nematode species and environmental conditions. They spend most of their active life within plant roots. Four juvenile stages in addition to egg-laying adult female line the success of their life cycle. The hatching second-stage juvenile (J2) in the soil are attracted by the plant root, burrow into the host root close to the growing tip, and migrate intercellular to reach the vascular cylinder. They establish and maintain permanent feeding cells in the host root to supply them with nutrients. Feeding cell formation requires a reprograming of the selected root vascular cells into specialized hypertrophied and hypermetabolic feeding cells. Successive nuclear divisions without cell division and isotropic growth lead to multinucleate feeding “giant cells” containing up to 100 endoreduplicated and hypertrophied nuclei (Favery et al., 2016). The induction of these giant cells, which are found only in RKN parasitism, is mediated by effector proteins secreted into the host tissues by the nematode (Truong et al., 2015; Nguyen et al., 2018; Vieira and Gleason, 2019). These effectors are produced principally in the nematode esophageal glands (one dorsal and two subventral glands) and are delivered to the plant *via* a syringe-like stylet. Some effectors may also be produced by other secretory organs such as the chemosensory amphids or the hypodermis (Zhao et al., 2019). Effectors may be secreted into the apoplast (i.e., the intercellular space) to facilitate root penetration, intracellular migration, and the suppression of host defenses or into the cytoplasm of the 5–7 vascular cells destined to become the giant cells. The effectors involved in giant cells neo-organogenesis and functioning may target different subcellular compartments and cellular host functions. Indeed, transcriptomic studies of the plant response to RKN infection have revealed that RKN can hijack several key host cellular processes, such as the cell cycle, phytohormone signaling, intercellular transport, and metabolism to favor parasitism (Caillaud et al., 2008; Gheysen and Mitchum, 2019; Favery et al., 2020).

*Meloidogyne incognita* secretes hundreds of effectors into host plants (Bellafiore et al., 2008; Wang et al., 2012). A role in parasitism has been demonstrated for very few of these effectors, and the target in the plant has been identified for only 10 of these proteins (Mejias et al., 2019, 2020; Zhao et al., 2020). RKN effectors often target multifunctional proteins regulating key biological processes and conserved in all eukaryotes. The known target proteins include annexins, which are involved in development and responses to the biotic and abiotic environment in plants (Baucher et al., 2012) and have been shown to interact with the *M. incognita* MiMIF-2 effector (Zhao et al., 2019). The *M. chitwoodi* Mc1194 effector targets a papain-like cysteine protease (PLCP; Davies et al., 2015). In plants, PCLPs are involved in physiological processes as diverse as seed germination, leaf senescence, abiotic stress responses, and immunity (Liu et al., 2018). Another example is provided by the PASSE-MURAILLE effector (MiPM), which has been shown to interact with the *Arabidopsis thaliana* CSN5 protein (Bournaud et al., 2018), a subunit of the COP9 signalosome (CSN), a multifunctional eukaryotic protein complex (Cope and Deshaies, 2003). Finally, the nuclear MiEFF18 targets the *Arabidopsis* core spliceosomal protein SmD1 (Mejias et al., 2020), which regulates pre-mRNA splicing and alternative splicing, thereby increasing the diversity of the giant cell proteome (Elvira-Matelot et al., 2016; Mejias et al., 2020).

We describe here the identification and functional analysis of the direct targets of *M. incognita* EFFECTOR 1 (MiEFF1), the first RKN effector shown to be secreted *in planta* and to target the nuclei of the feeding cells within the host giant cells (Jaouannet et al., 2012). *MiEFF1* (*Minc17998*) is specifically expressed in the dorsal esophageal gland of parasitic juveniles. However, MiEFF1 displays no similarity to any of the sequences present in database and has no domain of known function other than a predicted nuclear localization signal (NLS). Using the yeast two-hybrid (Y2H) approach and *in planta* bimolecular fluorescence complementation (BiFC), we demonstrated that, in *A. thaliana*, MiEFF1 interacts with multifunctional cytoplasmic proteins, a universal stress protein (AtUSP) and cytosolic glyceraldehyde-3-phosphate dehydrogenases (AtGAPCs) displaying moonlighting or having alternative nuclear functions required for plant responses to abiotic and/or biotic stress (Zaffagnini et al., 2013; Chi et al., 2019). We also showed that the *AtUSP* and *AtGAPC* genes are induced upon RKN parasitism. Finally, we demonstrated that *AtGAPCs* are required for *Arabidopsis* susceptibility to *M. incognita*.

## Materials and Methods

### Plant Material and Growth Conditions

The *Arabidopsis* lines used for the experiments were from the Wassilewskija (WS-4) and Columbia (Col-0) genetic backgrounds. The *usp* mutants, the SALK_146059 mutant line, referred to here as *usp.1*, previously described by Jung et al. (2015) and SALK_071209, referred to here as *usp.2*, were purchased from the *Arabidopsis* Biological Resource Center (United States). The *gapc1* (SALK_010839) and *gapc2* (SALK_016539) mutant lines and the *AtGAPC1* transcriptional reporter GUS line have been described elsewhere (Vescovi et al., 2013). The *abp39* and *abx27* mutants, harboring T-DNA insertions in *AtGAPC1* and *AtGAPC2*, respectively, were obtained from the INRAE Versailles T-DNA insertion collection (Bechtold et al., 1993). Homozygous mutants were identified by PCR-based genotyping, and new lines were further analyzed by RT-PCR and RT-qPCR, with the primers listed in Supplementary Table 1. Seeds of wild-type *A. thaliana*, mutants, and transgenic lines were surface-sterilized and sown on Murashige and Skoog (Duchefa) agar plates (0.5 × MS salts, 1% sucrose, 0.8% agar, and pH 6.4) or on a mixture of soil and sand. Seeds were incubated at 4°C for 2 days, and the plates were then transferred to a growth chamber with an 8 h photoperiod at 21°C. For propagation and transformation, seedlings were transferred to a growth chamber with a 16 h photoperiod at 21°C. *Nicotiana benthamiana* plants were grown in soil, under a 16 h photoperiod, at 24°C.

### Sequence Analysis and Alignment

The sequences of EFF1 orthologs were obtained from NCBI and Wormbase parasite. Protein sequences were aligned with the MAFFT tool on the EBI server.^1^

### Plasmid Constructs

The *M. incognita MiEFF1* (*Minc17998*; GenBank MW345915) and *MiEFF18* (*Minc18636*; GenBank KX907770) coding sequences (CDS) lacking the signal peptide, the *A. thaliana AtUSP* (*At3g53990*), *AtGAPC1* (*At3g04120*), *AtGAPC2* (*At1g13440*), and *AtSmD1b* (*At4g02840*), the *AtUSP* and *AtGAPC2* promoters, the SV40 T antigen and the murin P53 sequences were amplified by PCR with specific primers (Supplementary Table 1) and inserted into the pDON207 donor vector. The entry clones were recombined with pK7WGF2 (p35S:eGFP-GW), pK7WGR2 (p35S:RFP-GW), pKGWFS7 (GWpromoter:eGFP-GUS), BiFC vectors (pAM-35SS:GW-YFPc, pAM-35SS:GW-YFPn, pAM-35SS:YFPc-GW, and pAM-35SS:YFPn-GW) or Y2H vectors, pGBK-GW, pB27-GW, or pP6-GW (Karimi et al., 2002; Caillaud et al., 2009; Zhao et al., 2020), with Gateway technology (Invitrogen). All constructs were sequenced (GATC Biotech) and used to transform *Agrobacterium tumefaciens* strain GV3101 or *Saccharomyces cerevisiae* strains AH109, L40ΔGal4, or Y187.

### Y2H Assay

The yeast-two hybrid (Y2H) screen was performed according to the instructions supplied with the BD Matchmaker Library Construction and Screening Kit (Clontech). The coding sequence of MiEFF1 was inserted into the bait vector pGBKT7, which was used to transform *S. cerevisiae* strain AH109. For screening, cells carrying the bait construct were cotransformed with the commercial “normalized *A. thaliana* universal P02403 cDNA library” constructed by Dualsystems Biotech (Switzerland), using RNAs extracted from different *Arabidopsis* tissues, mixed in equal quantities and covering >95% of *Arabidopsis* genes. Yeasts displaying an interaction were recovered on selective SD medium without leucine, tryptophan, histidine, and adenine (SD/−Leu-Trp-His-Ade). To further validate the interactions, the *MiEFF1* CDS lacking the peptide signal was cloned into the bait pB27 vector and used to transform yeast strain L40ΔGal4. The whole sequences encoding the AtUSP, AtGAPC1, and AtGAPC2 were cloned into the pP6 prey vectors, which were used to transform yeast strain Y187. The pairwise mating experiments with Y187 (mat*α*) and L40ΔGal4 (mata) yeast strains were performed as previously described (Zhao et al., 2020).

### *In planta* Subcellular Localization and BiFC

Leaves from 3–4-week-old *N. benthamiana* plants were subjected to agroinfiltration with recombinant strains of *A. tumefaciens* containing GFP, RFP, or BiFC vectors, as described by Caillaud et al. (2009). Images were captured, 48 h after agroinfiltration, with an inverted confocal microscope (Zeiss LSM880) equipped with an argon and HeNe laser as the excitation source. Samples were excited at 488 nm for GFP/YFP and 543 nm for RFP. GFP or YFP emission was detected selectively with a 505–530 nm band-pass emission filter. We detected RFP fluorescence with a 560–615 nm band-pass emission filter.

### RNA Methods

Total RNA was extracted from *A. thaliana* seedlings or hand-dissected root segments and galls 7 and 14 days post-infection (dpi) with TRIzol reagent (Invitrogen), according to the manufacturer’s instructions. We reverse-transcribed 1 μg DNAse-treated (Ambion) RNA with the Superscript IV reverse transcriptase (Invitrogen). Semi quantitative RT-PCR was performed to analyze transcript abundancy in wild-type *Arabidopsis* and in the various newly generated mutant lines, with the primers described in Supplementary Table 1. Transcripts of the constitutively expressed *OXA1* gene (*At5g62050*) were amplified to check that the amounts of intact cDNA used were similar in RT-PCR experiments. RT-qPCR analyses were performed and analyzed as described by Mejias et al. (2020), with the primers described in Supplementary Table 1. *OXA1* and *UBP22* (*AT5G10790*) were used for the normalization of RT-qPCR data. Data were analyzed using the 2^-∆∆Ct^ method and were presented as Log2 fold change or as Normalized Relative Quantity generated using qBase software (Hellemans et al., 2007). Three technical replicates for 2–3 independent biological experiments were performed.

### Nematode Infection Assays

*Meloidogyne incognita* strain “Morelos” was multiplied in tomato (*Solanum lycopersicum* cv. “Saint Pierre”) grown in a growth chamber (25°C and 16 h photoperiod). Freshly hatched J2s were collected as previously described (Caillaud and Favery, 2016). Three-week-old *Arabidopsis* plants grown individually in small pots of soil/sand were inoculated with 150 *M. incognita* J2 larvae per plant. Roots were collected and weighted 6 weeks after infection, and the females laying egg masses stained with 4.5% eosin under a binocular microscope were counted. For each experiment, we used *n* = 17–25 plants per line. Four independent infection experiments were performed for each *gapc* mutant line, and one infection experiment was performed for each *usp* mutant line.

### Transgenic GUS Reporter Lines and Histochemical Analysis

The *AtGAPC1promoter::GUS* construct was previously described (Vescovi et al., 2013), and the *Arabidopsis* line was a kind gift from Prof. Alex Costa. The *AtGAPC2promoter::GUS* and *AtUSPpromoter::GUS* constructs were generated and introduced in *A. tumefaciens* as described above. *Arabidopsis thaliana* plants were transformed with those constructs by the floral dip method (Clough and Bent, 1998). Homozygous T3 transformants were used for further analysis. Plants were inoculated with *M. incognita* as described above, and GUS activity was analyzed histochemically 7 and 21 days after inoculation with *M. incognita*, as previously described (Caillaud et al., 2009). Reporter *GUS* expression in galls was revealed following 2–16 h staining. Two to four independent biological experiments were performed.

## Results

### MiEFF1 Interacts With a Universal Stress Protein and Cytosolic GAPDHs in *Arabidopsis*

**Figure 1:**
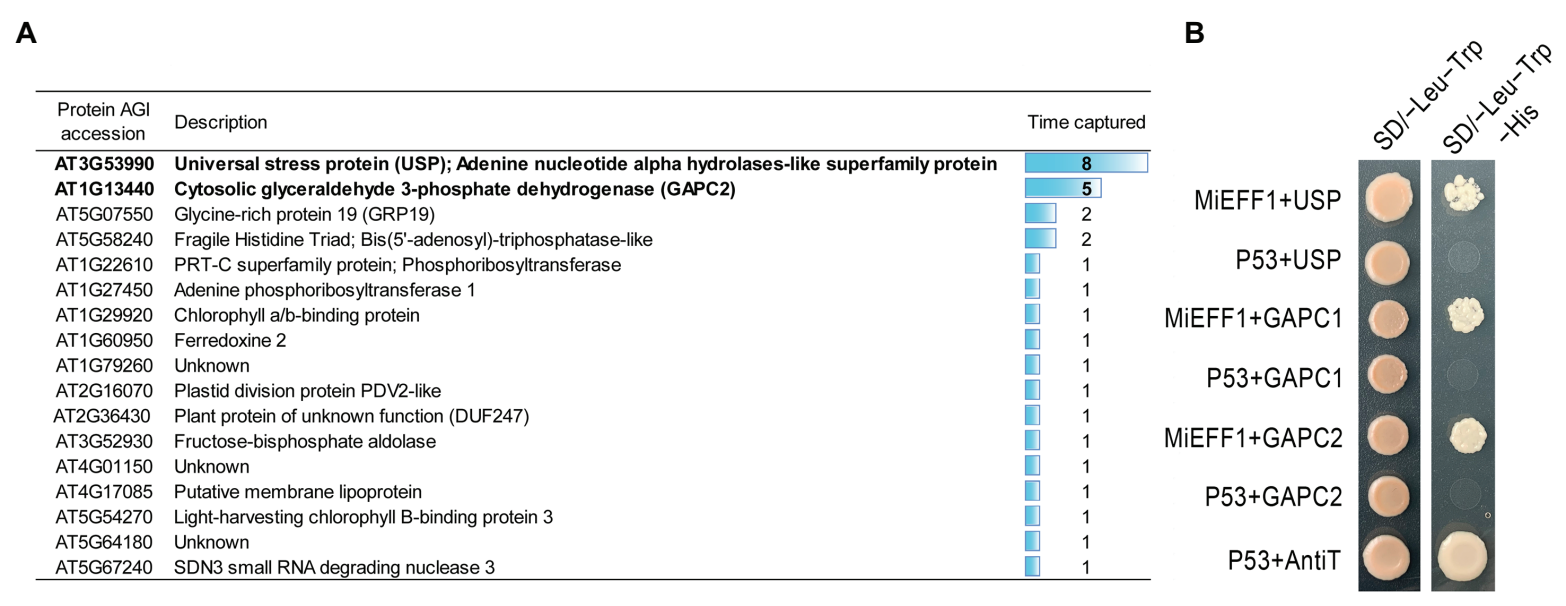
MiEFF1 interacts with AtUSP, AtGAPC1, and AtGAPC2 in yeast. **(A)** List of putative MiEFF1-interacting proteins from *Arabidopsis* obtained in our Y2H screen. **(B)** Yeasts containing the bait and prey plasmids carrying MiEFF1 and AtUSP, AtGAPC1, or AtGAPC2 and controls were spotted onto plates. SD/−Leu-Trp (Leu, leucine; Trp, tryptophane) is the non-selective medium without leucine and tryptophan. Only yeasts carrying a protein-protein interaction can survive on SD/−Leu-Trp-His (His, histidine) selective medium. Murine p53 and SV40 T antigen (anti T) were used as a positive control. Murine P53 and AtUSP, AtGAPC1, or AtGAPC2 were used as a negative control.

MiEFF1 is a pioneer protein of unknown function. Orthologs can be identified in the eight available RKNs genome sequences: *M. incognita*, *M. javanica*, *M. arenaria* (Blanc-Mathieu et al., 2017), *M. hapla* (Opperman et al., 2008), *M. enterolobii* (Koutsovoulos et al., 2020), *M. floridensis* (Lunt et al., 2014), *M. luci* (Susič et al., 2020), and *M. graminicola* (Phan et al., 2020; Supplementary Figure 1). EFF1 is, however, absent from other plant parasitic nematodes and other organisms. We searched for possible host targets of the MiEFF1 effector, by screening a normalized *Arabidopsis* prey Y2H library. Approximately 1.2 × 10^5^ yeast cells transformed with the prey and bait vectors were obtained, and 48 clones carrying a potential target were selected on SD/−Leu-Trp-His-Ade. Bait vector sequencing identified 30 sequences in frame with the transcriptional activation domain of GAL4 (Figure 1A). These sequences include that for the universal stress protein AtUSP (AT3G53990), which was captured eight times. All the clones isolated carried the complete sequence of *AtUSP* encoding a 160-amino acid (aa) protein containing the USPA domain (PF00582). In addition, five sequences encoding a cytosolic glyceraldehyde 3-phosphate dehydrogenase (AtGAPC2, AT1G13440) were identified. AtGAPC2 is 338 aa protein containing a NADP binding domain (aa 6–156, PF00044) and a catalytic domain (aa 161–318, PF02800). Two proteins were captured twice: a Glycine Rich Protein (AtGRP19, AT5G07550) and the Fragile Histidine Triad (AT5G58240) that are not nuclear proteins, and thus are not expected to interact with the MiEFF1 *in vivo* (Figure 1A). Finally, 13 proteins were captured once, that were not retained for further analysis (Figure 1A). Thus, only AtUSP and AtGAPC2 proteins were considered as potential targets of MiEFF1 in *A. thaliana*. We were also interested in analyzing whether AtGAPC1 (encoded by *At3g04120*), which is 97.9% identical to AtGAPC2 (Vescovi et al., 2013), could also interact with MiEFF1. For the confirmation of interactions, full-length sequences of *AtGAPC1*, *AtGAPC2*, and *AtUSP* were inserted into prey vectors to test the interactions with MiEFF1 in a LexA-based Y2H assay. The growth of diploids on selective SD/−Leu-Trp-His medium confirmed that the interactions between MiEFF1 and the full-length AtUSP, AtGAPC2, and AtGAPC1 proteins occurred in yeast (Figure 1B). These results demonstrate that MiEFF1 interacts with AtUSP, AtGAPC1, and AtGAPC2 in yeast.

### MiEFF1 Interacts With AtUSP, AtGAPC1, and AtGAPC2 in the Plant Cell Nucleus

**Figure 2:**
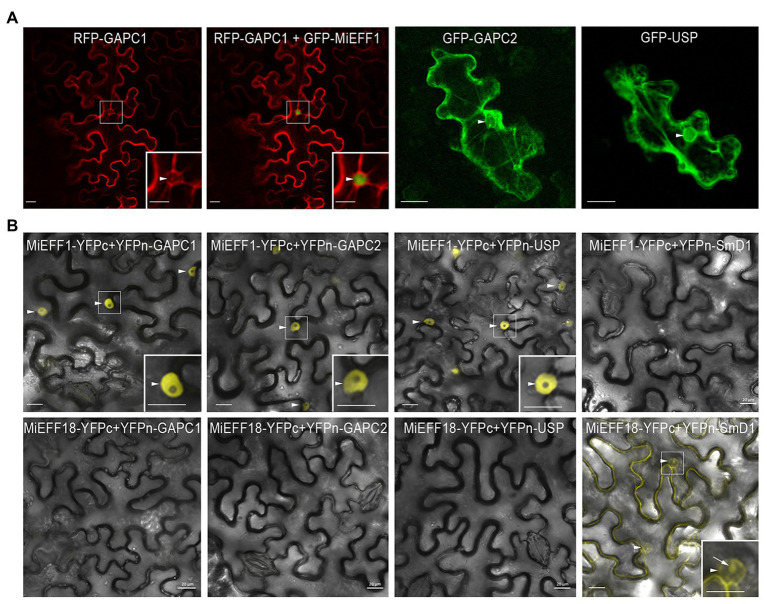
MiEFF1 interacts with AtUSP, AtGAPC1, and AtGAPC2 *in planta*. **(A)** Localization of GFP-AtUSP, -AtGAPC1, and -AtGAPC2 in *Nicotiana benthamiana* epidermal leaf cells. Enlargements of the area framed are shown. Arrowheads indicate nuclei. **(B)** MiEFF1 interacts with AtUSP, AtGAPC1, and AtGAPC2 in the nucleus in *Nicotiana benthamiana* cells. Confocal images of YFP fluorescence in bimolecular fluorescence complementation (BiFC) experiments with MiEFF1-YFPc and YFPn-GAPCs/USP fusion proteins expressed in *Nicotiana benthamiana* epidermal cells. MiEFF18 and AtSmD1b were used as controls. Three independent experiments were performed with similar results. Enlargements of the area framed are shown. Arrowheads indicate nuclei, arrow indicates nucleolus. Bars = 20 μm.

We investigated whether MiEFF1 would be able to interact physically with AtUSP and AtGAPCs in plant cells, by determining the subcellular distributions of these proteins *in planta*. We expressed the AtUSP, AtGAPC1, and AtGAPC2 proteins, fused to GFP or RFP, in *N. benthamiana* epidermal leaf cells, by agroinfiltration. AtGAPC1, AtGAPC2, and AtUSP were localized to the cytoplasm and the nucleus of agroinfiltrated *N. benthamiana* epidermal leaf cells (Figure 2A), consistent with the results obtained in *Arabidopsis* stable transgenic lines or protoplasts (Vescovi et al., 2013; Schneider et al., 2018), and in *Nicotiana tabacum* agro-infiltrated leaves (Melencion et al., 2017). These results suggest that the nuclear MiEFF1 (Figure 2A), and its identified targets would easily be able to interact in the nucleus of plant cells. We then performed BiFC to check the interactions between Mi-EFF1 and the AtGAPCs and AtUSP *in planta*. The co-expression of MiEFF1-YFPc and YFPn-GAPCs or YFPn-USP fusion proteins reconstituted YFP fluorescence signals in the nucleus of agro-infiltrated *N. benthamiana* epidermal cells (Figure 2B). Analogous combinations with the nuclear effector MiEFF18 (Mejias et al., 2020) as negative controls resulted in no interaction with any of the identified targets of MiEFF1 (Figure 2B). Similarly, no interaction was observed between MiEFF1-YFPc and the previously described nuclear protein AtSmD1b (Mejias et al., 2020), fused to an N-terminal YFPn (Figure 2B). Various other constructs, resulting in fusions with YFPc or YFPn at the C- or N-terminus of the encoded protein, were used to check the interactions between MiEFF1 and the three interacting proteins identified; all gave results similar to those presented in Figure 2B (Supplementary Figure 2). These results confirm that a specific interaction occurs between MiEFF1 and AtUSP, AtGAPC1, and AtGAPC2 in plant cells.

### AtGAPC and AtUSP Accumulate in RKN-Induced Feeding Sites

**Figure 3:**
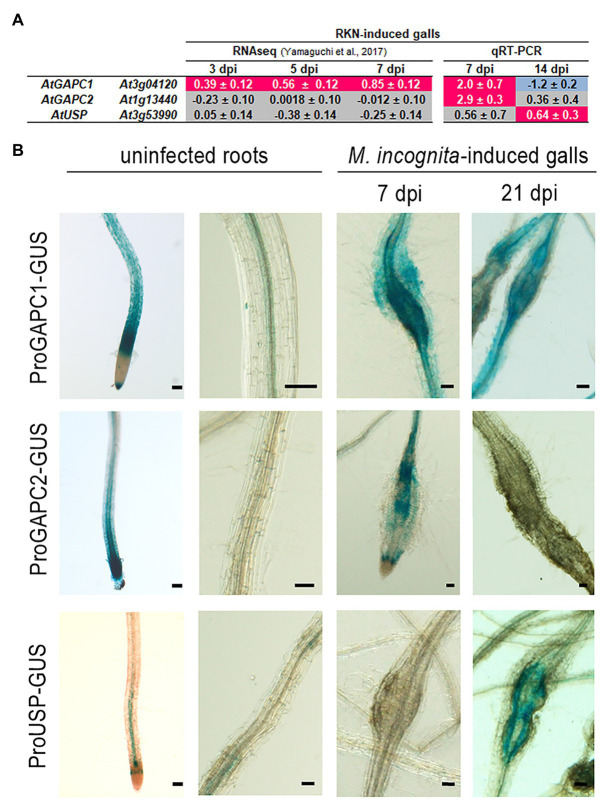
MiEFF1-interacting proteins accumulate in RKN-induced feeding sites. **(A)** Expression ratios of the *AtUSP*, *AtGAPC1*, and *AtGAPC2* genes in galls vs. uninfected roots (log2) were obtained from RNAseq (Yamaguchi et al., 2017) data. Data presented are log2 fold changes ± standard deviation (SD) from three biological replicates. The mRNA expression level of these genes was further measured by quantitative real-time PCR (RT-qPCR) in galls at 7 and 14 days post *M. incognita* infection (dpi) and uninfected roots. Data were normalized against *OXA1* and *UBP22* as constitutive genes. Data presented are log2 fold changes ± SD from two biological replicates. dpi, days post-infection. Gray coloring indicates an absence of differential expression, a magenta coloring indicates an upregulation and a blue coloring a downregulation. **(B)** GUS expression in galls at 7 and 21 dpi, and uninfected roots, of plants transformed with *AtGAPC1*, *AtGAPC2*, and *AtUSP* promoter –GUS fusions. Reporter *GUS* expression in galls was revealed following 2 h (*AtGAPC1promoter::GUS*) or 16 h (*AtGAPC2promoter::GUS* and *AtUSPpromoter::GUS*) staining. Two to three independent experiments were performed with similar results. NI, not infected. Bars = 50 μm.

We then investigated the expression of *AtUSP*, *AtGAPC2*, and *AtGAPC1* in giant cells and galls, using published transcriptomic data (Yamaguchi et al., 2017). These RNAseq data showed induction of *AtGAPC1* gene in galls at 3, 5, and 7 dpi (Figure 3A), suggesting a possible role for *AtGAPC1* in the plant response to nematode infection. No accumulation of AtGAPC2 and AtUSP was observed in galls according to those RNAseq data (Figure 3A). The expression pattern of *AtGAPC1*, *AtGAPC2* and *AtUSP* in galls, at 7 and 14 dpi, was further investigated using RT-qPCR. As shown in Figure 3A, *AtGAPC1* and *AtGAPC2* transcripts accumulated significantly in galls at 7 dpi. *AtUSP* expression increased slightly only at 14 dpi.

*Arabidopsis* transgenic lines carrying *promoter::GUS* constructs were used to analyze *AtGAPC1*, *AtGAPC2*, and *AtUSP* spatio-temporal expression upon *M. incognita* infection. As previously described (Vescovi et al., 2013), *AtGAPC1* is expressed in roots of non-infected plants (Figure 3B; Supplementary Figures 3, 4). Consistent with our RT-qPCR data, the transgenic plants carrying the *AtGAPC1promoter::GUS* construct (Vescovi et al., 2013) displayed strong promoter activity in galls at 7 dpi and to a lesser extent at 21 dpi (Figure 3B). The *AtGAPC2promoter::GUS* construct was generated by cloning a 1,200 bp sequence immediately upstream from the translation initiation codon. Transgenic plants carrying the *AtGAPC2promoter::GUS* construct displayed promoter activity in non-inoculated root tips (Figure 3B; Supplementary Figures 3, 4). Induction of *AtGAPC2* was observed in galls only at 7 dpi (Figure 3B), validating the transcriptomic data. No activity of the *AtGAPC2* promoter was detected at 21 dpi. Moreover, an *AtUSPpromoter::GUS* construct was generated, harboring the 1,070 bp sequence immediately upstream from the translation initiation codon. The *AtUSP* promoter displayed activity in vascular tissues of non-inoculated root tips (Supplementary Figure 5). No GUS signal was detected in galls at 7 dpi, but a signal was observed in galls at 21 dpi (Figure 3B; Supplementary Figures 3, 4). Thus, these results showed that *AtGAPC1* and *AtGAPC2* transcripts accumulated significantly in galls at 7 dpi, while *AtUSP* is expressed at a later stage of the interaction. Altogether, these data support the hypothesis that MiEFF1-interacting proteins may be involved in plant responses to RKN parasitism.

### AtGAPC1 and AtGAPC2 Are Involved in Plant Susceptibility to Root-Knot Nematodes

**Figure 4:**
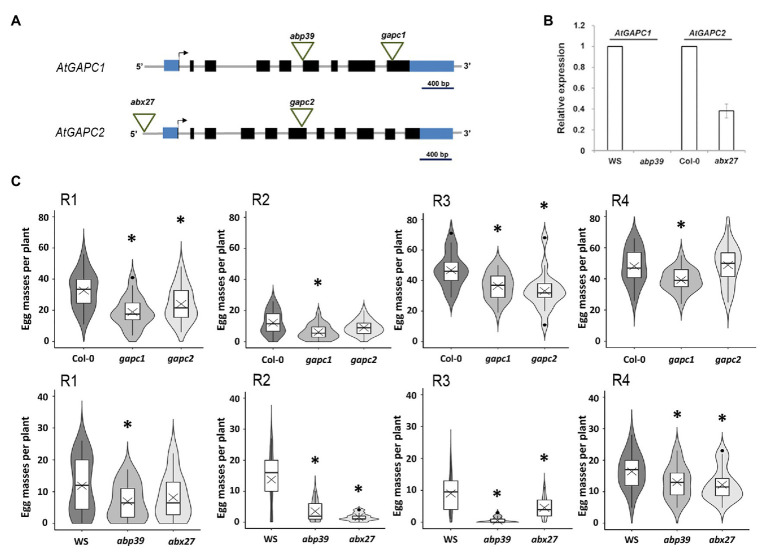
*AtGAPC1* and *AtGAPC2* are involved in *Arabidopsis* susceptibility to *M. incognita*. **(A)** Schematic illustration of the genomic organization of *AtGAPC1* and *AtGAPC2* and T-DNA insertion sites. Black boxes represent exons, gray lines correspond to introns, blue boxes represent untranslated sequences and the arrows represent START codons. **(B)** RT-qPCR analysis of *AtGAPC1* and *AtGAPC2* expression in *abp39* and *abx27* mutants. Data were normalized against *OXA1* and *UBP22* constitutive genes. **(C)** Results of four independent nematode infection assays, performed on *Arabidopsis* mutants *AtGAPC1* (*gapc1* and *abp39*) and *AtGAPC2* (*gapc2* and *abx27*), relative to wild-type plants (Col-0 and WS). Number of egg masses (*y*-axis), at 6 weeks post infection, in independent nematode infection assays, is shown as violin-plot diagrams. Box indicates interquartile range (25–75th percentile). The horizontal bar in the box indicates the median of the reported values. The crosses show the mean value. Whiskers mark the lowest and highest values within 1.5 times the interquartile range, and black dots indicate outliers. The box plot is included in a kernel density plot (shade of grays) showing the entire distribution of the data. R1–R4 indicate independent replicates. *n* = 17–25 plants per line. Asterisks indicate a significant difference between the wild-type and the mutant lines, as shown by Student’s *t* test (*p* < 0.05).

We further investigated the possible involvement of the proteins encoded by the *AtUSP*, *AtGAPC1*, and *AtGAPC2* genes in the development and/or physiology of the giant cells induced by RKN, by performing infection tests with *M. incognita* in *Arabidopsis* loss-of-function mutant lines. A previously characterized *usp.1* knockout (KO) line (SALK_146059; Jung et al., 2015) and a new *usp.2* KO mutant line (SALK_071209; Supplementary Figure 5) carrying a T-DNA insertion in the third exon were challenged with *M. incognita*. We scored the number of females producing egg masses 6 weeks after infection. The test of these two alleles indicated no significant difference in the number of females producing egg masses between the *usp* mutants and Col-0 wild-type plants (Supplementary Figure 5). The two previously described *gapc1* and *gapc2* KO lines in the Col-0 genetic background (Vescovi et al., 2013; Figure 4A) were used here to investigate the role of *AtGAPC* genes in the plant-RKN interaction. We also selected two new mutant alleles in the WS genetic background, KO *abp39* and knockdown *abx27*, carrying T-DNA insertions in the sixth exon of *AtGAPC1*, and in the *AtGAPC2* promoter, respectively (Figures 4A,B; Supplementary Figure 6). These lines were inoculated with *M. incognita* juveniles. For all the lines tested, the number of females producing egg masses was smaller in the mutants than in wild-type plants: 19.4–94.0% fewer egg masses were found in *atgapc1* mutants and 25.7–88.3% fewer in *atgapc2* mutants (Figure 4C). A slight root developmental phenotype was observed in the *gapc2* mutant, but not in the *abx27* (Supplementary Figure 6), and both these *gapc2* mutant lines exhibited a rather similar decreased susceptibility to RKN. For the two *atgapc1* mutants, a significant and reproducible reductions in the egg mass number were observed over four independent experiments (Figure 4C), and this phenotype is not associated to a defect in root development (Supplementary Figure 6). Overall, these results demonstrate that *AtGAPC* genes play a role in *Arabidopsis* susceptibility to RKN.

### AtGAPC1 and AtGAPC2 Regulate Expression of Defense-Associated Genes

**Figure 5:**
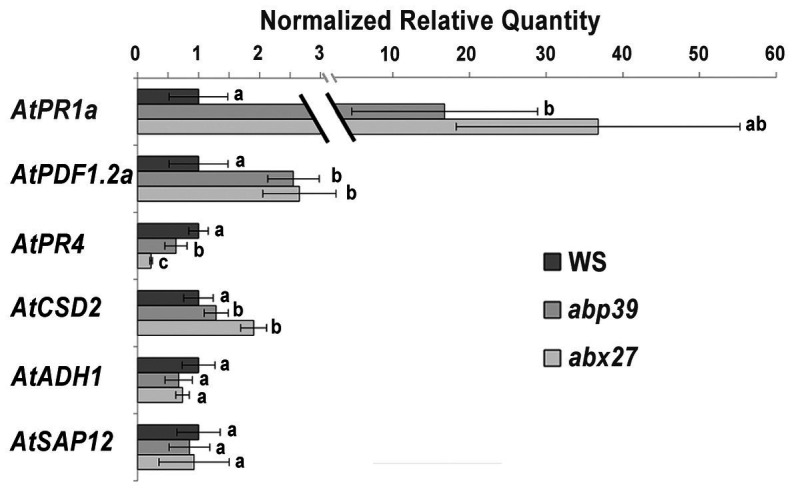
AtGAPC1 and AtGAPC2 regulate expression of defense-associated genes in Arabidopsis. RT-qPCR was used to investigate expression of defense- and stress-associates genes in *abp39* and *abx27* mutant seedlings. The genes considered were *AtPR1a* (*At2g14610*; salicylic acid (SA)-mediated defense response marker gene), *AtPDF1.2a* (*At5g44420*; encoding ethylene- and jasmonate-responsive plant defenses), *AtPR4* (*At3g04720*; ethylene-responsive pathogenesis-related protein), *AtCSD2* (*At2g28190*; chloroplastic Cu/Zn superoxide dismutase), *AtADH1* (*At1g77120*; catalyzing the reduction of acetaldehyde with NADH as reductant), and *AtSAP12* (*At3g28210*; stress-associated protein). *AtOXA1* (*AT5G62050*) and *AtUBP22* (*AT5G10790*) were used as internal controls. Data are presented as means ± standard deviation (SD) from three biological replicates. Different letters indicate statistically significant differences, *p < 0.05, Wilcoxon sign-rank test.

To investigate whether AtGAPC1 and AtGAPC2 manipulation by MiEFF1 could affect *Arabidopsis* immunity, we measured, using RT-qPCR, the expression of genes involved in antioxidative functions (*AtCDS2*) and abiotic (*AtADH1* and *AtSAP12*) or biotic (*AtPR1a*, *AtPDF1.2a*, and *AtPR4*) stress responses (Zhao et al., 2020) in *abp39* and *abx27* mutants. In both mutants, *AtPR1a* and *AtPDF1.2a*, regulators of salicylic acid (SA)- and ethylene- and jasmonate (JA)-mediated defense responses, respectively, showed a strong constitutive expression, while *AtPR4*, encoding an ethylene-responsive PR protein, was repressed in both lines (Figure 5). The *AtCDS2* gene encoding a Cu/Zn superoxide dismutase was also significantly induced in both mutants while the *AtADH1*, *AtSAP12* encoding an alcohol dehydrogenase and a stress-associated protein, respectively, were not differentially expressed (Figure 5). These results indicate that targets of MiEFF1 are involved in regulating the expression of SA and JA defense-related genes in *Arabidopsis*.

## Discussion

Plant parasitic nematodes of the genus *Meloidogyne* have developed original and complex mechanisms of parasitism. By injecting proteins called as “effectors” into the host plant, they induce cellular reprograming and the transformation of root cells into hypertrophied polynucleate feeding cells known as “giant cells.” The development and maintenance of giant cells induced by RKN requires the manipulation of several host cellular processes (Favery et al., 2016). The nucleus is a key cellular compartment that must be targeted by the parasite (Quentin et al., 2013), and effectors, such as Mi16D10 and MiEFF18, have already been shown to target host transcription factors and the splicing machinery, respectively (Huang et al., 2006; Mejias et al., 2020). MiEFF1, a protein carrying a NLS, is produced in the parasitic RKN dorsal gland, secreted *in planta* and has been shown to accumulate in host giant cell nuclei during parasitism (Jaouannet et al., 2012). However, the function of MiEFF1 remained unknown. The identification of pathogen effector targets is now widely used as an approach to elucidating the molecular functions of these effectors (Khan et al., 2018; Mejias et al., 2019). Our yeast two-hybrid experiments identified three potential targets of the MiEFF1 nuclear effector in *Arabidopsis*: AtUSP, and two cytosolic GAPDHs, AtGAPC1 and AtGAPC2.

USPs were originally discovered in *Escherichia coli* and have been implicated in responses to various stress conditions in bacteria, archaea, plants, and some invertebrate animals (Vollmer and Bark, 2018). Plant USPs are multifunctional proteins involved in the development in addition to their role in responses to biotic and abiotic stresses (Chi et al., 2019). USPs proteins containing the *E. coli* universal stress protein A (USPA) domain and are encoded by members of a multigene family in *Arabidopsis* (Kerk et al., 2003; Chi et al., 2019). AtUSP (AT3G53990) has been implicated in plant responses to drought (Isokpehi et al., 2011), oxidative stress (Jung et al., 2015), and heat and cold stress (Jung et al., 2015; Melencion et al., 2017). A role in plant defense against pathogens was recently documented, with AtUSP accumulating in plants exposed to *Pseudomonas syringae* and displaying antimicrobial activity against various fungi (Park et al., 2017). We confirmed the presence of AtUSP in both the cytoplasm and the nucleus (Melencion et al., 2017), consistent with a possible dual function for this protein. In the cytoplasm, AtUSP acts as a protein chaperone in response to heat shock (Jung et al., 2015; Chi et al., 2019), whereas, in the nucleus, it may bind and protect nucleic acids, particularly RNA, enabling the plant to tolerate cold stress (Melencion et al., 2017; Chi et al., 2019). The induction of *AtUSP* upon RKN attack, at the end of giant cell formation, and its interaction with MiEFF1 within the plant cell nucleus suggest that it is the nuclear function of AtUSP in nucleic acid protection that is targeted by MiEFF1. Further investigation will be required, using multiple KO lines because of the redundancy of *USPs* in *A. thaliana* (Kerk et al., 2003), to conclude on AtUSP function in plant-RKN interaction.

Interestingly, we also found that nuclear MiEFF1 interacted with two cytosolic GAPDHs. Not only these proteins act as key enzymes in glycolysis, but are also well-known “moonlighting” (multifunctional) proteins with functions in several processes unrelated to metabolism in animal and plant cells, such as apoptosis, autophagy, gene expression regulation, and responses to abiotic or biotic stress (Sirover, 2011; Zaffagnini et al., 2013). This functional versatility is regulated, in part, by redox-based post-translational modifications that alter GAPDH catalytic activity and influence the subcellular distribution of the enzyme. Animals have a single isoform of GAPDH, with well-established moonlighting properties, whereas plants have multiple isoforms, the non-metabolic roles of which have yet to be discovered (Zaffagnini et al., 2013). *Arabidopsis* has four gene families encoding seven phosphorylating GAPDH isoenzymes and one non-phosphorylating GAPDH isoenzyme. Only AtGAPC1 and AtGAPC2 and the non-phosphorylating GAPDH (NP-GAPDH) are cytosolic. The other GAPDHs are located in other cellular compartments such as chloroplasts (Zaffagnini et al., 2013). Interestingly, the two cytoplasmic GAPCs have been shown to relocalize to the plant cell nucleus upon exposure to oxidative stresses, such as H_2_O_2_ (Schneider et al., 2018) or nitric oxide (Testard et al., 2016; Schneider et al., 2018), and following exposure to cadmium (Vescovi et al., 2013), salt stress (Wawer et al., 2010), or heat stress (Kim et al., 2020). Similarly, a strong accumulation of AtGAPC1 has been shown in the nucleus following the perception of flagellin during infection with *P. syringae*, and a mutation of *AtGAPC1* renders *Arabidopsis* less susceptible to these pathogenic bacteria (Henry et al., 2015). The function of GAPCs in the nucleus remains unclear. They have been shown to interact with plant nucleic acids, suggesting a potential role of GAPCs in protecting nucleic acids during stress responses (Testard et al., 2016). GAPCs were recently shown to interact with the NF-YC10 transcription factor to promote the expression of heat-inducible genes and heat tolerance in *Arabidopsis* (Kim et al., 2020). We show here that *AtGAPC1* and *AtGAPC2* were induced during giant cell formation and that the corresponding proteins were targeted by the nuclear effector MiEFF1, suggesting that one of the moonlighting nuclear functions of these proteins is targeted by RKN. A stronger and faster response of *AtGAPC1* was observed in response to *M. incognita*, in line with the response to biotic and abiotic stress previously described (Vescovi et al., 2013; Henry et al., 2015). We also found that GAPCs were important for parasitic success in RKNs, as shown for several bacterium-host and virus-host interactions (Han et al., 2015; Henry et al., 2015; Zeng et al., 2018). Finally, *N. benthamiana* GAPCs have been shown to be targeted by the citrus tristeza virus (CTV), *via* interaction with the viral p23, to facilitate the infectious cycle of the virus (Ruiz-Ruiz et al., 2018). Thus, GAPCs appear to be common targets of evolutionarily diverse plant-pathogens. Considering *AtGAPC1* induction in galls, and susceptibility of *gapc1* mutants to *M. incognita*, we postulate that AtGAPC1 assume most of GAPCs function required for disease establishment. GAPC2 would reinforce the action of GAPC1 at a key moment of giant cell formation. Previous studies showed GAPCs have multiple functions in the regulation of autophagy, hypersensitive response, and plant innate immunity, and described a role of GAPCs as negative regulators of plant defense in *Arabidopsis* (Henry et al., 2015), *N. benthamiana* (Han et al., 2015), or *Manihot esculenta* (Zeng et al., 2018). Microarray analysis on *Arabidopsis gapc* mutants showed that several genes encoding for enzymes regulating reactive oxygen species (ROS) homeostasis, such as peroxidases, catalases, or superoxide dismutases, had altered expression (Rius et al., 2008), and here, using a RT-qPCR approach, we demonstrated a differential expression of defense- and stress-associated genes in *abp39* and *abx27* mutants, both mutant lines showing strong constitutive expression of *AtPR1a* and *AtPDF1.2a* genes.

While several RKN effectors were shown to be responsible for host defense suppression (Quentin et al., 2013; Vieira and Gleason, 2019), very few have had their host target identified, i.e., MiMIF2 (Zhao et al., 2019), MiPDI1 (Zhao et al., 2020), Mg16820 (Naalden et al., 2018), MgMO237 (Chen et al., 2018), and MjTTL5 (Lin et al., 2016). This work adds MiEFF1 to the list of RKN effectors modulating host immunity and confirms that defense- and stress-related proteins are key targets of effectors, particularly those of nematodes, during disease establishment. These genes, particularly those encoding GAPCs, constitute attractive conserved candidates for targeting to reduce susceptibility in novel breeding strategies aiming to develop durable and broad-spectrum resistance (van Schie and Takken, 2014; Zaidi et al., 2018).

## Data Availability Statement

The original contributions presented in the study are included in the article/Supplementary Material, further inquiries can be directed to the corresponding authors.

## Author Contributions

NT designed and performed the experiments and interpreted the results. YC contributed to BiFC and Y2H experiments. JM contributed to subcellular localizations. SSo and KM performed the qRT-PCR analysis. MJ contributed to material and data analysis. NT, PA, BF, and MQ wrote the article. SJ-P, SSa, PA, BF, and MQ obtained the funding, designed the work, supervised the experiments, and data analyses. All authors contributed to the article and approved the submitted version.

### Conflict of Interest

The authors declare that the research was conducted in the absence of any commercial or financial relationships that could be construed as a potential conflict of interest.

The handling editor declared a past co-authorship with one of the authors PA.

## Footnotes

^1^https://www.ebi.ac.uk/Tools/msa/mafft/
