# Supplementary material for: The Meloidogyne incognita Nuclear Effector MiEFF1 Interacts With Arabidopsis Cytosolic Glyceraldehyde-3-Phosphate Dehydrogenases to Promote Parasitism
Source: Front Plant Sci. 2021 Apr 9;12:641480. doi: 10.3389/fpls.2021.641480 (PMC8062903; doi:10.3389/fpls.2021.641480)
Supplement: Supplementary file 1 [file Data_Sheet_1.PDF]

Frontiers in Plant Science

Article title: The *Meloidogyne incognita* nuclear effector MiEFF1 interacts with *Arabidopsis* cytosolic glyceraldehyde-3-phosphate dehydrogenases to promote parasitism

Authors: Nhat My Truong, Yonpan Chen, Joffrey Mejias, Salomé Soulé, Karine Mulet, Maelle Jaouannet, Stéphanie Jaubert-Possamai, Shinichiro Sawa, Pierre Abad, Bruno Favery, Michaël Quentin

The following Supporting Information is available for this article:

Supplemental table 1 | Primers used in this study.

Supplemental Figure 1 | EFF1 is a conserved and specific RKN effectors.
Supplemental Figure 2 | MiEFF1 interacts with AtUSP, AtGAPC1 and AtGAPC2 in the nucleus in *N. benthamiana* cells.
Supplemental Figure 3 | ATH1 array expression profiles of *AtGAPC1, AtGAPC2* and *AtUSP* genes.
Supplemental Figure 4 | Characterization of *AtGAPC1, AtGAPC2* and *AtUSP* promoter activity patterns in Arabidopsis roots.
Supplemental Figure 5 | Functional analysis of Arabidopsis USP in response to *M. incognita.*
Supplemental Figure 6 | Molecular analysis and phenotype of the Arabidopsis *gapc* mutants used in this study.

Supplemental Table 1 | Primers used in this study.

Name	Sequence (5'- 3')	Purpose
MiEFF1	AAAAAGCAGGCTTCACCATGACAATCGTTAGGCGTGAATCG	Gateway cloning entry vector
MiEFF1	AGAAAGCTGGGTGTTATTTTCGTTCAGGAATTGCAC	
MiEFF18	AAAAAGCAGGCTTCACCATGGCTCGAACCATTTCTAATATGG	Gateway cloning entry vector
MiEFF18	AGAAAGCTGGGTGTTAATGCTTCTTTCCTCCTTTTG	
MiEFF16	AAAAAGCAGGCTTCACCATGAAGAATAACGACCACCATAAAC	Gateway cloning entry vector
MiEFF16	AGAAAGCTGGGTGTCATTCATCATCCCCACATTCAAC	
AtGAPC1_GW5	AAAAAGCAGGCTTCACCATGGCTGACAAGAAGATTAG	Gateway cloning entry vector
AtGAPC1_GW3_Stop	AGAAAGCTGGGTGTTAGGCCTTTGACATGTGGACG	
AtGAPC2_GW5	AAAAAGCAGGCTTCACCATGGCTGACAAGAAGATCAG	Gateway cloning entry vector
AtGAPC2_GW3_Stop	AGAAAGCTGGGTGTTAGGCCTTTGACATGTGAACG	
AtGAPC2_GW3	AGAAAGCTGGGTGGGCCTTTGACATGTGAACG	
AtUSP_GW5	AAAAAGCAGGCTTCACCATGCCTAAAGACAGGAATATCG	Gateway cloning entry vector and RT-PCR
AtUSP_GW3_Stop	AGAAAGCTGGGTGTTATTCGTTATCCTTGACAACG	
AtUSP_GW3	AGAAAGCTGGGTGTTCGTTATCCTTGACAACG	Gateway cloning entry vector
USPpromoter-F	AAAAAGCAGGCTTCACCTGATCATACCAAGCAAAGCAGTCC	Gateway cloning entry vector and genotyping of the two *usp* mutants
USPpromoter-R	AGAAAGCTGGGTGCTTTCTTGACCCTTTTTTTCTTCTC	
GAPC2promoter-F	AAAAAGCAGGCTTCACCGGAGGAGAAGAAGAAGACTTTTGGC	Gateway cloning entry vector
GAPC2promoter-R	AGAAAGCTGGGTGCGAAATTGAGATCGAGAGAGATTTAG	
AtSmD1_GW5	AAAAAGCAGGCTTCACCATGAAGCTCGTCAGGTTTTTGATG	Gateway cloning entry vector
AtSmD1b_GW3_Stop	AGAAAGCTGGGTGCTAACGACCTCTGCCGCGACCAC	
murine p53_GW3_STOP	AGAAAGCTGGGTCGGGATGCAGAGGCAGTCAGTC	Gateway cloning entry vector
murine p53_GW5	AAAAAGCAGGCTCCCCTGTCACCGAGACCCCTGGG	
SV40 T-antigen_GW3_STOP	AGAAAGCTGGGTCTGTTTCAGGTTCAGGGGGAGG	Gateway cloning entry vector
SV40 T-antigen_GW5	AAAAAGCAGGC TCC GGAACTGATGAATGGGAGCAG	
AttB1	GGGGACAAGTTTGTACAAAAAAGCAGGCT	Analysing Gateway destination vectors
AttB2	GGGGACCACTTTGTACAAGAAAGCTGGGT	
AttL1	TCGCGTTAACGCTAGCATGGATCTC	Analysing Gateway entry vectors
AttL2	GTAACATCAGAGATTTTGAGACAC	
P35S	GATGACGCACAATCCCACTATC	Sequencing Gateway destination vectors
T35SM	CTACTCACACATTATTCTGGAG	
YFPnF	GAGGGCGAGGGCGATGCCACCTACG	Analysing Gateway destination vectors (BiFC)
YFPcF	CTTCAAGATCCGCCACAACATCG	
YFPnR	CCGTCGTCCTTGAAGAAGATGGT	Analysing Gateway destination vectors (BiFC)
YFPcR	CGAACTCCAGCAGGACCATGTG	
DNA-BD	TTTTCGTTTTAAAACCTAAGAGTC	Amplification pGBG bait vector
3'AD	AGATGGTGCACGATGCACAG	Amplification pGAD prey vector
T7	TACGACTCACTATAGGGC	Amplification and sequencing pGAD and pGBG vectors
MiEFF1-sfi-noATG	GGGGCCGGACGGGCCACAATCGTTAGGCGTGAATCGGT	Cloning in pB27
MiEFF1-sfi-Stop	AGGGGCCCCAGTGGCCTTATTTTCGTTCAGGAATTGCA	
LexA	GTCAGGTCGTTGTCGCACGT	Sequencing bait vector pB27
terminator tADH1	CGACCTCATGCTATACCTGAGA	
pP6-F	GCCTCCTCTAACGTTCAT	Sequencing prey vector pP6
pP6-R	GCGGGGTTTTTCAGTATC	
AtGAPC1_At3g04120_E4L	CCTACATGTTCAAGTACGACAGTG	Genotyping of the *abp39* mutant and RT-PCR
AtGAPC1_At3g04120_E8R	CTGACAGTAAGGTCAACAACTGAG	Genotyping of the *abp39* mutant
AtGAPC2_At1g13440L	TTGCTGTCACATTCCATCTGAC	Genotyping of the *abx279* mutant
AtGAPC2_At1g13440R	ATCGAGAGAGATTTAGATGATATCG	
TAG5	CTACAAATTGCCTTTTCTTATCGAC	Genotyping of the *abp39* and *abx27* mutants
gapc1_L	CCGCACATCTGTTAATGAATTTC	Genotyping of the *gapc1* mutant
gapc1_R	CTCAGAAGACTGTTGATGGGC	
gapc2_L	GGTTAGGACTGAGGGTCCTTG	Genotyping of the *gapc2* mutant
gapc2_R	GGCATCAGGTACATAATCATGG	
LBa1_Salk	TGGTTCACGTAGTGGGCCATCG	Genotyping of the *gapc1*, *gapc2* and *usp* mutants
OXA1_F	TACCTGATCTGCCTCCACCT	housekeeping gene qRT-PCR
OXA1_R	AACAGGACTCAGCGATGTTG	
UBP22_F	GCCAAAGCTGTGGAGAAAAG	housekeeping gene qRT-PCR
UBP22_R	TGTTTAGGCGGAACGGATAC	
gapc1-RT_L	GAGGTGATGGGAGTTTGTAGAC	RT-PCR and qRT-PCR
gapc1-RT_R	TACGTCATCATCAACGGG	qRT-PCR
gapc2-RT_L	GCTTACACCGGCGAGAGTTTGTG	RT-PCR
gapc2-qRT_L	TGCGCAGTCATGAGAGTT	qRT-PCR
gapc2-RT_R	AGTTGCCAGTTGGGTTTG	RT-PCR and qRT-PCR
usp-RT_L	TATCGGAATCGCCATGGATT	qRT-PCR
usp-RT_R	TCTCGATCGCCCATTTCAG	
AtPR1a_F	CGGAGCTACGCAGAACAACT	qRT-PCR
AtPR1a_R	CTCGCTAACCCACATGTTCA	
AtPDF1.2a_F	TGGTGGAAGCACAGAAGTTG	qRT-PCR
AtPDR1.2a_R	GATCCATGTTTGGCTCCTTC	
AtPR4_F	TAGTGGACCAATGCAGCAAC	qRT-PCR
AtPR4_R	GATCAATGGCCGAAACAAG	
AtCSD2_F	CACGGAGCTCCAGAAGATGAG	qRT-PCR
AtCSD2_R	CGGCATTGGCATTTATGTTTC	
AtADHl_F	GGGAGAGAGTATTCGTTGCATCA	qRT-PCR
AtADHl_R	GTGAACATCATCTGCGAGAGAATG	
AtSAP12_F	ATGGCAGGAGGAGGAACAG	qRT-PCR
AtSAP12_R	CTAAAACGATCTAACTGATGGGTGAAGA	

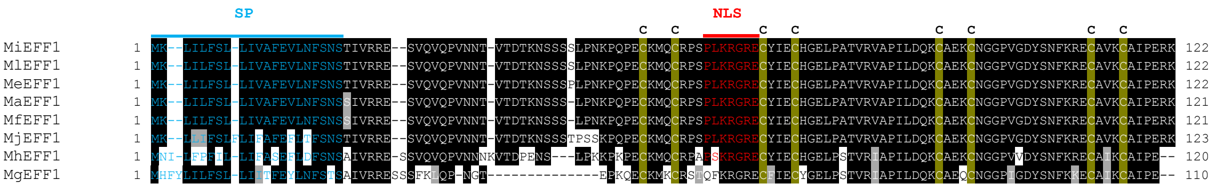

Supplemental Figure 1 | EFF1 is a conserved and specific RKN effectors. Protein sequence alignment (MAFFTtool, EBIserver) of the closest MiEFF1 homologs in sequenced RKN species. *M. incognita* MiEFF1 (Minc17998, Minc3s01352g23040, MW345915), *M. luci* MlEFF1 (M. luci contig000032F), *M. enterolobii* MeEFF1 (Ment3s00208g0245161, CAD2172942), *M. arenaria* MaEFF1 (Mare1s00544g011508), *M. floridensis* MfEFF1 (maker-nMf.1.1.scaf11715-augustus-gene-0.6-mRNA-1), *M. javanica* MjEFF1 (Mjav1s12336g064417), *M. hapla* MhEFF1 (MhA1_Contig138.frz3.gene15) and *M. graminicola* MgEFF1 (Mgra_00002554, KAF7638103). The blue line underlines the secretion signal peptide (SP) as predicted by SignalP3.0 (http://www.cbs.dtu.dk/services/SignalP-3.0/), the red line underline the conserved nuclear localization signal (NLS) as predicted by PSORTII (http://psort.hgc.jp/). Eight conserved cystein residues (C) predicted to be engaged in disulfide bounds are shown in brown. The alignment was colored using BoxShade.

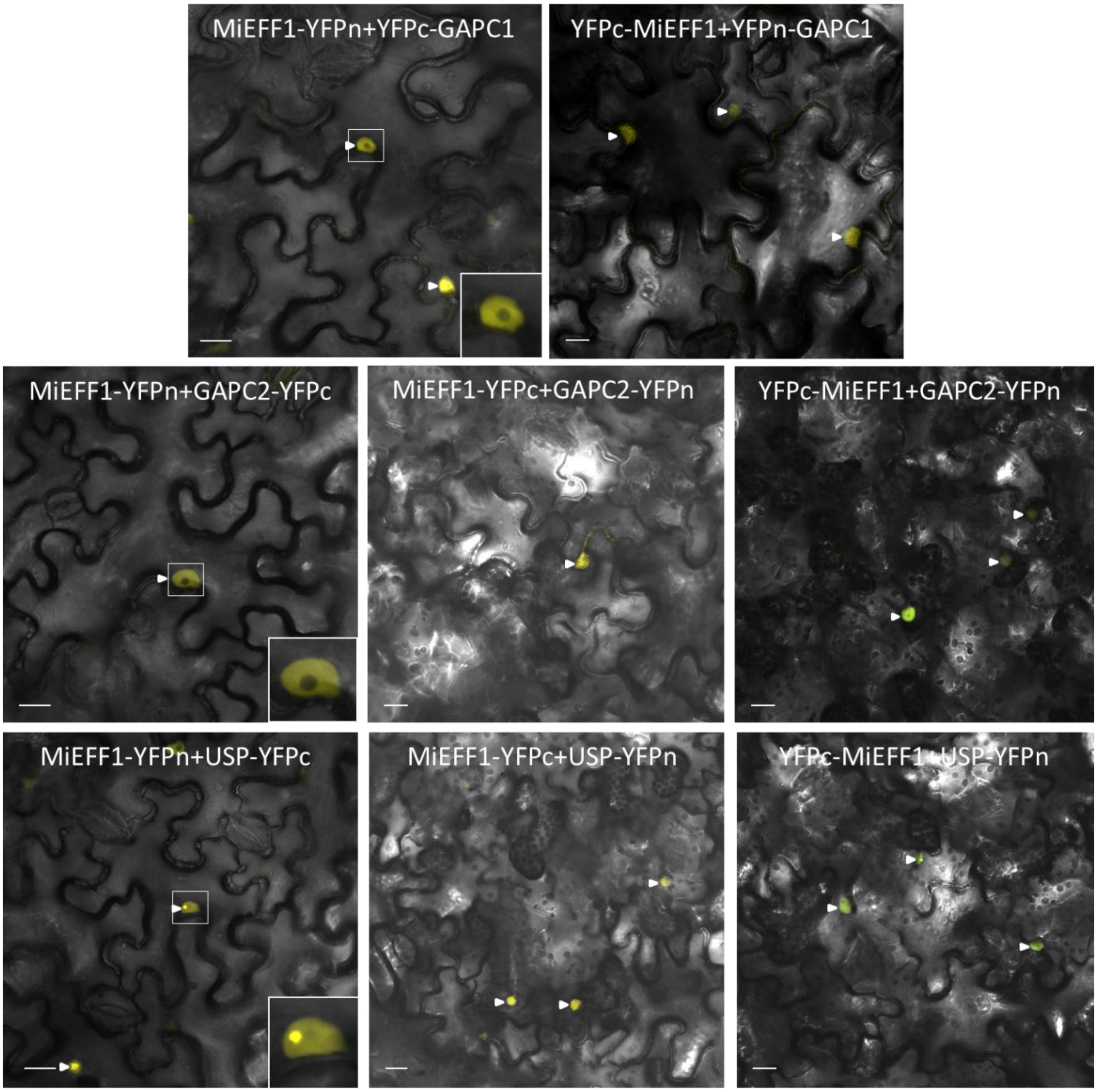

Supplemental Figure 2 | MiEFF1 interacts with AtUSP, AtGAPC1 and AtGAPC2 in the nucleus in *N. benthamiana* cells. Confocal images of YFP fluorescence in bimolecular fluorescence complementation (BiFC) experiments with different fusion proteins expressed in *N. benthamiana* epidermal cells. Enlargements of the area framed are shown. Arrowheads indicate nuclei. Bars = 20 µm.

At3g04120, AtGAPC1

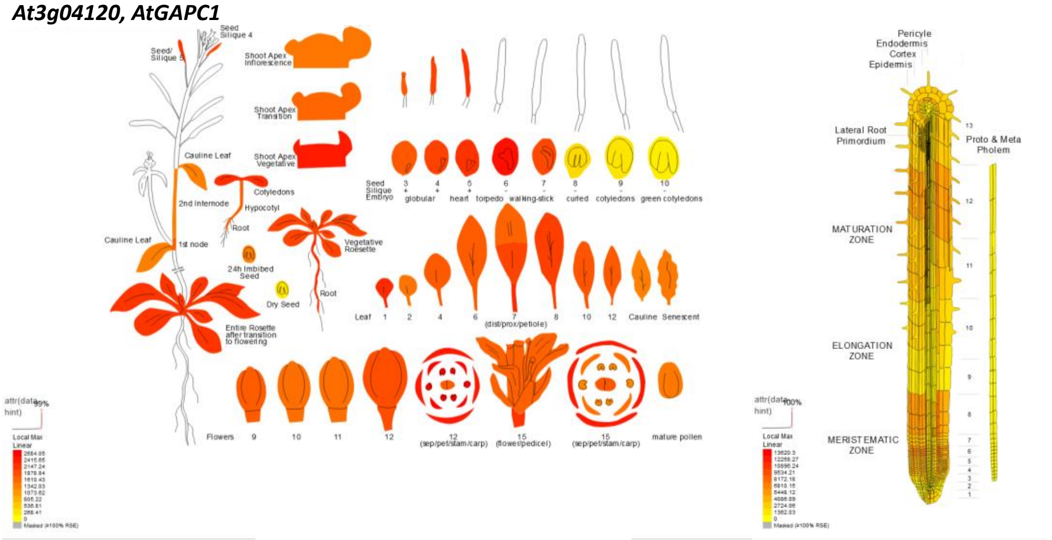

At1g13440, AtGAPC2

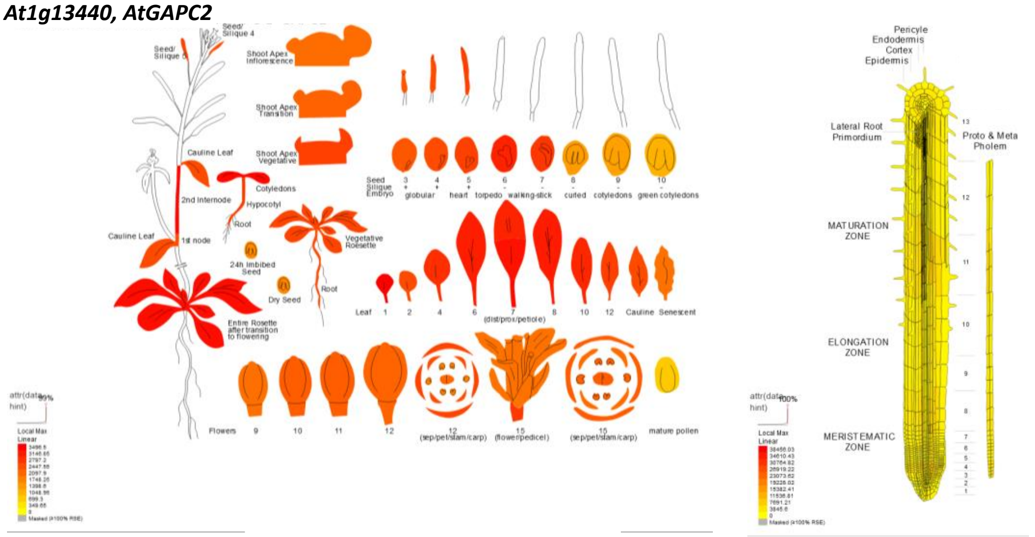

At3g53990, AtUSP

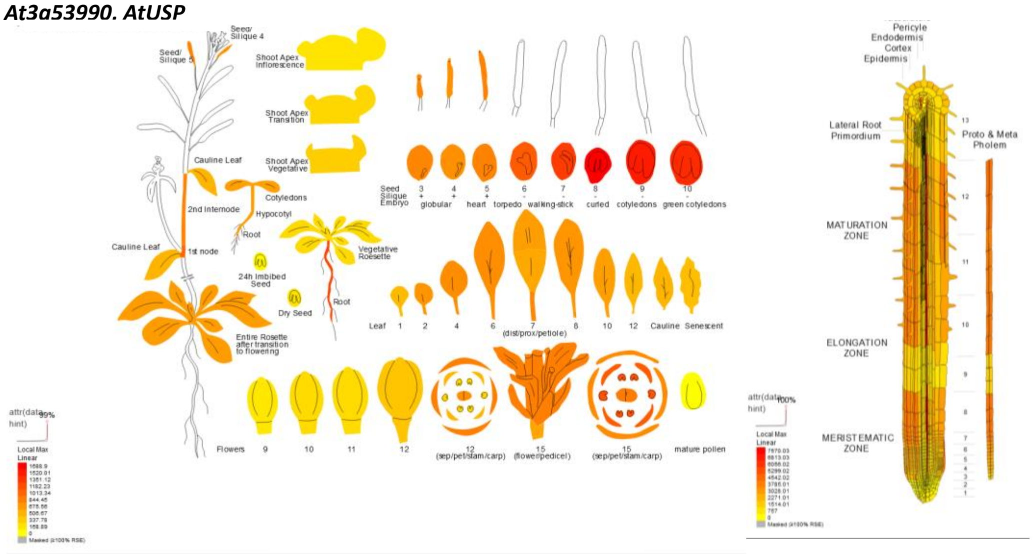

Supplemental Figure 3 | ATH1 array expression profiles of *AtGAPC1*, *AtGAPC2* and *AtUSP* genes. Expression data were retrieved from the Arabidopsis eFP Browser. The expression of each gene is shown in different tissues. Normalization methods, the tissue, and the developmental stages of each sample as well as additional information can be found at http://bar.utoronto.ca/eplant/.

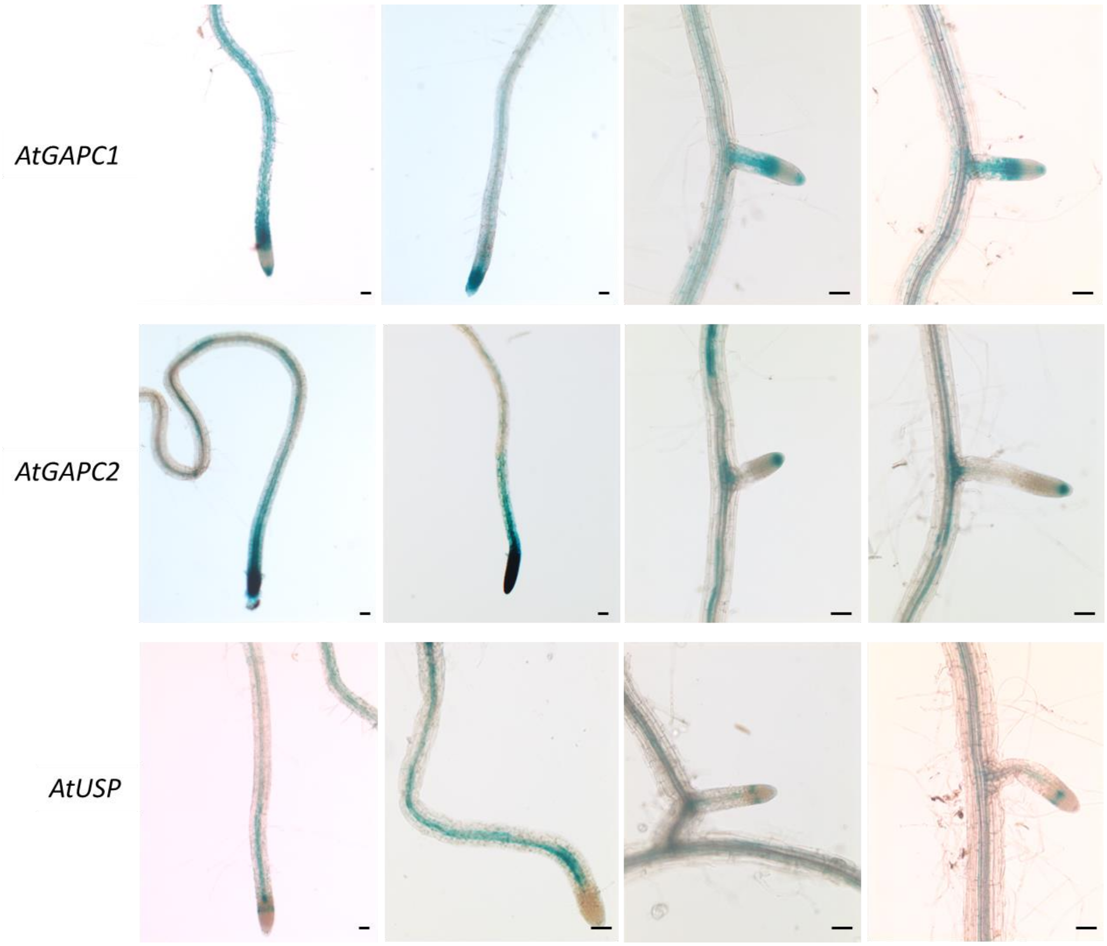

Supplemental Figure 4 | Characterization of *AtGAPC1, AtGAPC2* and *AtUSP* promoter activity patterns in Arabidopsis roots. Histochemical analysis of GUS activity in roots of 4-week-old *AtGAPC1promoter::GUS*, *AtGAPC2promoter::GUS* and *AtUSPpromoter::GUS* transgenic plants. Bars = 150 μm.

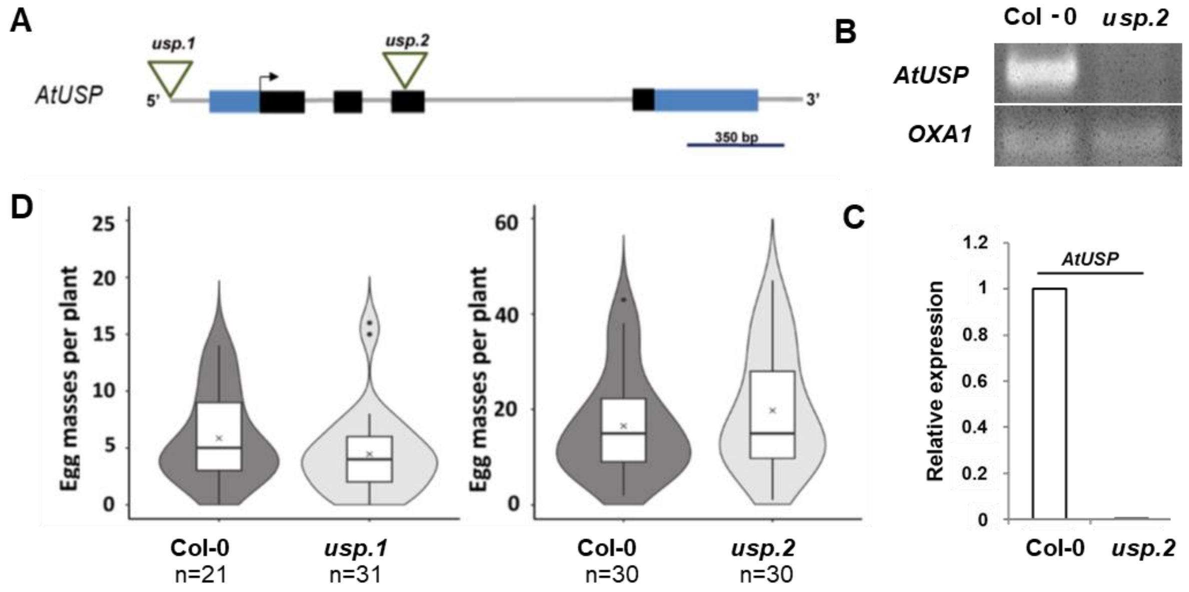

Supplemental Figure 5 | Functional analysis of *Arabidopsis USP* in response to *M. incognita*. **(A)** Schematic illustration of *AtUSP* genomic organisation and T-DNA insertion sites. Black boxes represent exons, grey lines correspond to introns, blue boxes represent untranslated sequences and the arrows represent START codons. **(B)** RT-PCR revealed absence of transcripts of the *AtUSP* gene in the *usp.2* mutant line and its presence in wild-type Col0 plants. Amplification of the constitutively expressed *OXA1* gene (At5g62050) transcript showed that similar amounts of intact cDNAs were used for RT-PCR experiments. **(C)** RT-qPCR analysis of *AtUSP* expression in *usp.2* mutants. Data were normalized against *OXA1* as constitutive gene; similar results were obtained when normalizing with UBP22. **(D)** Results of a nematode infection assay performed on Arabidopsis *usp* mutants (*usp.1* and *usp.2*) relative to wild-type Col-0 plants. The number of egg masses (y-axis) at 6 weeks post infection is shown as violin-plot diagrams. The number (n=21 to 31) of plants tested is indicated. Box indicates interquartile range (25th to the 75th percentile). The horizontal bar in the box indicates the median of the reported values. The crosses show the mean value. Whiskers mark the lowest and highest values within 1.5 times the interquartile range, and black dots indicate outliers. The box plot is included in a kernel density plot (shade of greys) showing the entire distribution of the data. No significant difference between the wild type and the mutant lines was detected by Student's t test ($p<0.05$).

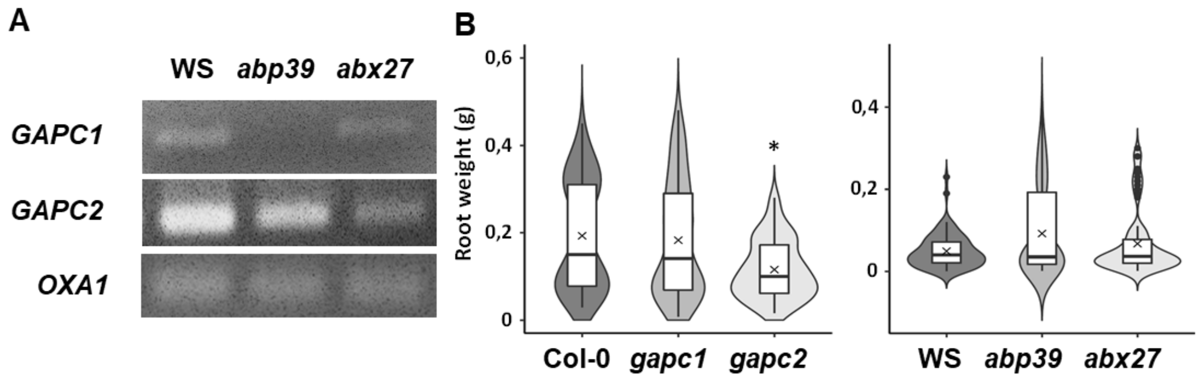

Supplemental Figure 6 | Molecular analysis and phenotype of the *Arabidopsis gapc* mutants used in this study. **(A)** RT-PCR revealed absence of *AtGAPC1* transcripts in the *abp39* mutant line and decrease amount of *AtGAPC2* transcripts in the *abx27* line compared to wild-type WS plants. Amplification of the constitutively expressed *OXA1* gene (At5g62050) transcript showed that similar amounts of intact cDNAs were used for RT-PCR experiments. **(B)** Arabidopsis phenotypes associated with the mutation of *AtGAPC* genes. Root weights of Arabidopsis mutant lines shown as violin-plot diagrams. Box indicates interquartile range (25th to the 75th percentile). The horizontal bar in the box indicates the median of the reported values. The crosses show the mean value. Whiskers mark the lowest and highest values within 1.5 times the interquartile range, and black dots indicate outliers. The box plot is included in a kernel density plot (shade of greys) showing the entire distribution of the data. For each line, we used n = 83 to 97 plants. Asterisks indicate a significant difference between the wild type and the mutant line, as shown by Student's *t* test (p<0.05).
